# Epithelioid Hemangioendothelioma of Spleen and Bone: A Case Report

**DOI:** 10.5334/jbsr.3600

**Published:** 2024-06-25

**Authors:** Melvin Denolf, Annelies Rappaport, Sigrun Delvaux

**Affiliations:** 1 Department of Radiology, Sint Trudo Hospital, Sint-Truiden, Belgium; 2 Department of Radiology, Sint-Trudo Ziekenhuis, Sint-Truiden, Belgium; 3 Department of Pathology, Sint-Trudo Hospital, Sint-Truiden, Belgium

**Keywords:** Epithelioid hemangioendothelioma, focal splenic lesion, CAMTA1

## Abstract

Epithelioid hemangioendothelioma (EHE) is a rare vascular tumor that can originate in various parenchymatous organs, soft tissue, and bone. Extrahepatic involvement is exceedingly rare. In this case, multifocal disease in the spleen and bone was present. Bone lesions showed a target appearance. Splenic lesions showed delayed enhancement of solid components with persistent rim enhancement. A bone biopsy with CAMTA1 staining confirmed the diagnosis.

*Teaching point:* The presence of multifocal bone and splenic lesions can raise suspicion of EHE when other multifocal diseases are excluded.

## Introduction

Epithelioid hemangioendothelioma is a rare vascular tumor, most commonly seen in the liver; extrahepatic involvement is relatively unknown to radiologists.

## Case History

A 48-year-old male patient without oncologic history was referred for a 99mTc single photon emission computed tomography (SPECT)/computed tomography (CT) examination of the lumbar spine due to lower back pain.

**Figure 1:**
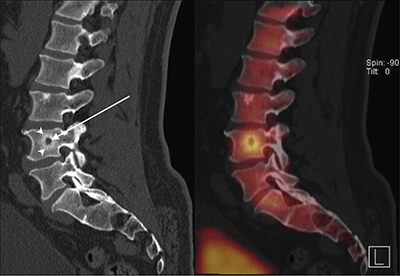
SPECT/CT lumbar spine.

SPECT-CT showed multiple lesions with a low attenuating center (Figure 1, arrow) and thick sclerotic rim (Figure 1, arrowheads) in the lumbar spine and pelvic bone with avid tracer uptake.

**Figure 2:**
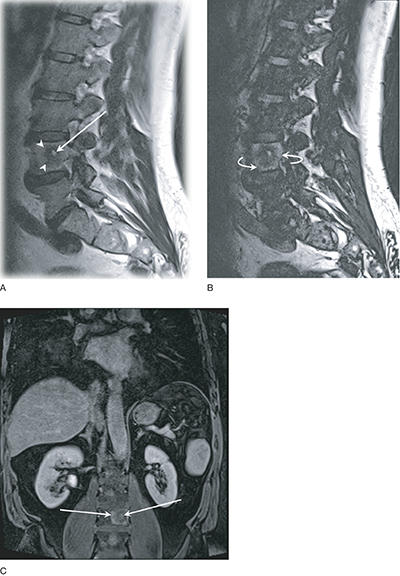
MRI lumbar spine. **(A)** T2 Dixon image-in phase; **(B)** T2 Dixon image-out phase; **(C)** T1 VIBE fat-saturated image (with contrast).

Magnetic resonance imaging (MRI) of the lumbar spine was performed. Lesions exhibited a target-like appearance, with a central T2-hyperintense core (Figure 2A, arrow), a surrounding thick T2-hypointense inner rim (Figure 2A, arrowheads) corresponding with sclerosis on CT and an outer rim of T2-hyperintensity (Figure 2B, curved arrows); without intralesional fat (Figure 2B). The lesion showed contrast enhancement of the core and outer layer (Figure 2C, arrows).

**Figure 3:**
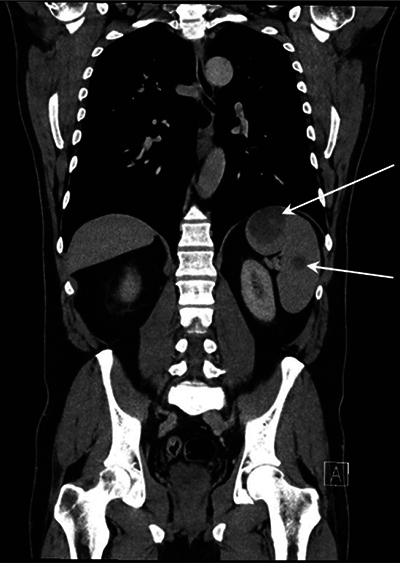
Thoracoabdominal CT.

Because of the presumed diagnosis of bone metastasis, a thoracoabdominal CT scan was performed, revealing multiple hypovascular nodular lesions in a slightly enlarged spleen (Figure 3, arrows) and without other organ involvement.

**Figure 4:**
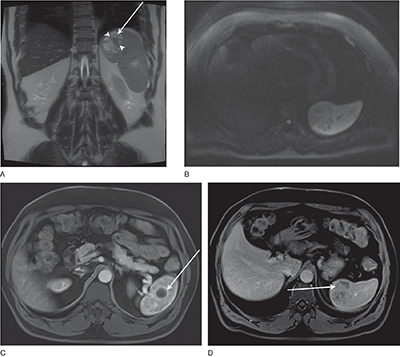
MRI spleen.

Subsequent splenic MRI showed T2-hyperintense lesions (Figure 4A, arrow) with central nodular T2-isointense components (Figure 4A, arrowheads) and isointense septations, without diffusion restriction (Figure 4B). Contrast administration showed rim enhancement (Figure 4C, arrow) throughout all the phases and delayed enhancement of the central nodular components (Figure 4D, arrow).

A CT-guided bone biopsy was performed for the final diagnosis. The pathological evaluation exhibited significant positivity for CAMTA1 rearrangement, confirming the diagnosis of epithelioid hemangioendothelioma (EHE).

## Discussion

### General features

EHE is a rare (1:1000000) low- to intermediate-grade malignant vascular tumor with clinical and histological features resembling angiosarcoma and hemangioma [1]. EHE can present at any age but is more frequent during the second and third decades [1]. Hepatic and pulmonary EHE show a female preponderance, whereas osseous EHE has a male preponderance [1].

### Imaging features

Bone involvement of EHE is often multifocal, occurs in the axial and appendicular skeletons and when multifocal, the lesions have the tendency to occur in bones of the same region, especially in the foot [2].

On X-ray and CT, lesions appear osteolytic with sporadically a sclerotic rim. On MRI, bone lesions are frequently T2-hyperintense, T1-iso/hypointense, with homogenous contrast enhancement, and may show a sclerotic rim [1, 2]. In this case, the bone lesions had a targetoid appearance, showing a similar targetoid T2-signal as described in hepatic EHE lesions, but lacking the avascular outer rim of hepatic EHE lesions, making this resemblance rather coincidental.

Splenic EHE lesions are well defined, non-targetoid nodules on CT and MRI with enhancement of the solid components. In the case presented, the solid components showed progressive contrast enhancement to become isointense to splenic parenchyma after approximately 5 minutes and showed persistent peripheral rim enhancement, characteristics that are also described in hepatic EHE [ 3 ]. On MRI, the nonsolid components appear T2-hyperintense and T1-hypointense, and the solid components appear T2- and T1-isointense. DWI characteristics of splenic EHE aren’t described in the literature. In this case, the solid components were isointense to the splenic parenchyma on diffusion weighted imaging (DWI) and apparent diffusion coefficient (ADC) map and the hypoenhancing components showed a low signal on DWI and high signal on ADC map, probably corresponding to hypocellular components.

### Differential diagnosis

Multifocal splenic and bone lesion in the absence of primary malignancy raise the suspicion of hematologic malignancy or vascular neoplasms.

Focal lesions in **lymphoma** show higher diffusion restriction and lower ADC-values than EHE. In lymphoma, the presence of lymphadenopathy is characteristic [3].

Bone lesions in **multiple myeloma** can appear similar to EHE, but multiple myeloma is appearing at an older age, and splenic involvement is rare.

**Splenic hemangiomas** are T2 hyperintense, can show heterogenous T2-appearance in larger lesions with the presence of centripetal contrast enhancement [3].

**Typical osseous hemangiomas** are T2-hyperintense, contain intralesional fat, and tend to show thickened trabeculae on CT. EHE bone lesions can be T2-hyperintense but lack other typical hemangioma features. Atypical bone hemangiomas can be difficult to differentiate from EHE.

**Cystic angiomatosis** is a rare disease presenting as cystic splenic and bone lesions, but atypical cases are described in the literature and can be difficult to differentiate from EHE [2].

**Multifocal/metastatic primary angiosarcoma** is difficult to differentiate from multifocal EHE, but angiosarcoma has a more aggressive course (bone destruction, ill-defined splenic lesions) and occurs at an older age [2, 3].

### Pathology

The histologic appearance of EHE ranges from well-differentiated to highly undifferentiated. The tumor contains vascular and stromal components. Genetically, CAMTA1 and WWTR1 were identified as two genes involved in the chromosomal translocation that is characteristic of EHE [1]. The identification of CAMTA1 rearrangement through FISH is highly specific for EHE and confirms the diagnosis.

## Conclusion

The atypical imaging features of extrahepatic involvement in EHE are challenging. Radiologists can suggest the diagnosis by recognizing certain imaging characteristics of EHE, excluding metastatic disease, and excluding typical features of other multifocal hematologic or vascular neoplasms. A biopsy remains necessary for the ultimate diagnosis.
